# Sema3A drastically suppresses tumor growth in oral cancer Xenograft model of mice

**DOI:** 10.1186/s40360-017-0163-4

**Published:** 2017-07-06

**Authors:** Chao Huang, Yi Wang, Jian-Hua Huang, Weixian Liu

**Affiliations:** 1 Department of Oral Surgery, Shengjing Hospital of China Medical University, Shenyang, China; 2 Department of Oral Surgery, First Affiliated Hospital of Liaoning Medical University, Jinzhou, 121000 China; 3 Department of Oral Surgery, Shengjing Hospital of China Medical University, Shenyang, 110001 China

**Keywords:** Sema3A, Angiogenesis, Oral cancer, Tumor growth

## Abstract

**Background:**

Multiple studies suggest anti-angiogenesis to be a promising and rational option in cancer treatment. Interestingly, the axonal sprouting inhibitor semaphorin 3A (Sema3A), a potent suppressor of tumor angiogenesis in various cancer models, is lowly expressed in human oral cancer. Thus, we hypothesized that overexpression of Sema3A in human oral cancer cells may have potential therapeutic effects.

**Methods:**

The LentiSema3A-EGFP was first constructed and transduced to the tongue squamous cell carcinoma cell line SSC-9. Angiogenesis assay was performed with endothelial cell tube formation assay and chorioallantoic membrane (CAM) assay. Tumor xenografts model was used to evaluate the effect of Sema3a on the tumor growth. Finally, western blot was performed to study the mechanisms of inhibiting angiogenesis by Sema3A.

**Results:**

In vitro and in vivo approaches revealed that Sema3A significantly inhibited tube formation of endothelial cells and reduced angiogenesis in CAM assay. In addition, overexpression of Sema3A in the tongue squamous cell carcinoma cell line SSC-9 resulted in significantly reduced angiogenesis and drastically suppressed tumor growth in mice. Mechanistically, Sema3A inhibited the phosphorylation of VEGFR2, as well as Src and FAK, downstream of the VEGF/VEGFR2 pathway.

**Conclusion:**

Our results demonstrated that overexpression of Sema3A in oral cancer cells drastically suppressed tumor growth by inhibiting angiogenesis. Our findings provide a basis for the development of novel therapeutics in the management of oral cancer.

## Background

Oral cancer is generally regarded as a malignant tumor occurring in the oral cavity. It accounts for 1.9–3.5% of systemic cancers, and 4.7%–20.3% of head and neck malignancies, ranking second to nasopharyngeal carcinoma among all head and neck cancers [1]. In the clinic, the treatment methods for oral cancer include surgery, radiation therapy, and chemotherapy; however, the results remain unsatisfactory, with a very high recurrence rate [2–4]. With the emergence of biotechnology methods such as gene therapy, immunotherapy, and cancer stem cell therapy, more opportunities are available for the treatment of oral cancer. Biological treatment can reduce the side effects that often occur with the traditional therapy, making it possible to improve the survival rate after treatment of oral cancer patients.

Angiogenesis is important in the growth, metastasis, and prognosis of malignant solid tumors [5]. It is necessary for further growth of malignant solid tumors after breaking through the epithelial basement membrane. Newly formed tumor blood vessels have unique structural characteristics: the wall is not complete, with no smooth muscle components; it is composed only of endothelial cells and the basal membrane [6]. These properties enable fast growth and promote distant tumor metastasis [7–9]. Thus, inhibition of angiogenesis is an important approach in treating malignant solid tumors [10].

Semaphorins (Semas) were first recognized as functional orientation axon guidance molecules, but there is growing evidence that the family members of semaphorins are closely related to tumor cell migration, tumor growth, immune response, angiogenesis, and a variety of physiological and pathological phenomena [11]. Semaphorins are expressed in a variety of organisms. According to structure and amino acid sequence, they can be divided into eight types: Sema1 and Sema2 are found in invertebrates, Sema3 ~ 7 exist in vertebrates, and Sema8 is encoded by viruses. Among them, Sema2 and Sema3 are secreted proteins, while the other members are membrane-associated molecules. Sema3, the only secreted protein of this kind in vertebrates [12, 13], inhibits angiogenesis [14–20].

Low Sema3A expression has been described in oral cancer tissues [21]. Therefore, its overexpression in oral cancer cells would have important clinical significance in inhibiting angiogenesis and tumor growth. In this study, by lentiviral-mediated overexpression of Sema3A in oral cancer cells, we assessed whether Sema3A decreases the growth of oral cancer by inhibiting angiogenesis, further exploring the mechanism of angiogenesis inhibition by Sema3A.

## Methods

Tongue squamous cell carcinoma (SCC-9 ATCC CRL-1629) cells and HUVEC (ATCC CRL-1730) were purchased from American Type Culture Collection (ATCC, USA). Nude mice were purchased from the Institute of Chinese Academy of Medical Sciences and maintained under a 12:12 h light-dark cycle at 20–25 °C. All animal work and experimental protocols were approved by the Ethics Committee for Animal Experiments of the China Medical University, strictly complying with the institutional guidelines and criteria outlined in the “Guide for Care and Use of Laboratory Animals”.

### Lentiviral vector construction

The lentiviral expression plasmid GV287 and packaging plasmids (Helper 1.0 and Helper 2.0) were purchased from Genechem (Shanghai, China). This system belongs to the third generation of lentiviral vector. The GV287 vector contains the basic components of HIV 5’LTR and 3’LTR as well as other secondary auxiliary elements. The Helper1.0 vector contains the gag gene of the HIV virus, which encodes the major structural protein of the virus; the pol gene, which encodes virus-specific enzyme; and the rev gene, which encodes the genes that regulate the expression of gag and pol gene. The Helper 2.0 vector contains the VSV-G gene from the herpes simplex virus, which provides the envelope protein required for viral packaging. The Sema3A gene was cloned and amplified using polymerase chain reaction (PCR) with the following primers: GAGGATCCCCGGGTACCGGTCGCCACCATGGGCTGGTTAACTAGGATTGTCTG and TCCTTGTAGTCCATACCGACACTCCTGGGTGCCCTCTCAAATTC; then, the gene was inserted into the AgeI site of GV287 to generate pGV287Sema3A. The pGV287Sema3A was transformed into *E.coli*, and the right clones were proved by PCR with the specific primers: AAATGGAGACCCACTGAC and CGTCGCCGTCCAGCTCGACCAG Recombinant lentiviral vectors were produced by co-transfecting 293 T cells with the lentiviral expression plasmid pGV287Sema3A and the packaging plasmids (Helper 1.0 and Helper 2.0). A total of 293 T cells (6 × 10^5^) were cultured in a 10 cm tissue culture plate with opti-MEM (GIBCO, USA). Transfection was performed when cell density reached 30–40% confluency. Plasmid GV287Sema3A and the packaging plasmids were co-transfected into 293 T cells using Lipofectamine 2000 according to the manufacturer’s instructions. 293 T cells were cultured routinely. After 6 h of culture, the medium was replaced by DMEM (GIBCO, USA). Infectious lentiviruses were harvested at 48 h post-transfection and concentrated. The viral titer was determined by the dilution gradient method and calculated as follows: virus titer (TU/ml) = (counted fluorescent cells/corresponding dilution time) × 100. Virus vectors were stored at −80 °C until use.

### In vitro lentiviral transduction

At 70% confluency, SCC-9 cells were infected with Lenti Sema3A-EGFP at 40 MOI. After 48 h, transduction efficiency was assessed by fluorescent microscopy. The expression of Sema3A in SCC-9 cells was detected after 48 h of transfection with LentiSema3A-EGFP by Western blot.

### Western blot

SCC-9 cells or tumor tissues were washed twice with cold PBS and resuspended in cold lysis buffer containing 20 mmol/L HEPES (pH 7.5), 150 mmol/L NaCl, 1 mmol/L EDTA, 0.5% Triton X-100, and protease inhibitors (Roche). Similar quantities of total protein (20 μg) were separated by SDS-PAGE, transferred onto nitrocellulose membranes, and blocked overnight in blocking solution at 4 °C. For Sema3A detection, the membranes were incubated for 1 h with anti-human Sema3A monoclonal antibodies (Santa Cruz Biotechnology), followed by anti-mouse secondary antibody conjugated to horseradish peroxidase (Zymed, Inc., South San Francisco, CA) for 1 h at room temperature. For the detection of phosphorylated VEGFR2, Src, and FAK, the membranes were incubated for 1 h with mouse monoclonal antibodies raised against human p-VEGFR2 (Invitrogen), p-Src (Invitrogen), and p-FAK (Cell Signaling), respectively, followed by anti-mouse or anti-rabbit secondary antibodies conjugated to horseradish peroxidase (Zymed, Inc., South San Francisco, CA) for 1 h at room temperature. The enhanced chemiluminescence ECL Plus system (Amersham Biosciences UK, Little Chalfont, UK) was used to reveal the signals. Beta-actin was used as an internal control.

### Tube formation assay

Tube formation assay was performed on growth factor reduced matrigel coated plates. Briefly, 250 μl cold matrigel was coated on 24 well plates, and incubated at 37 °C for 1 h for polymerization. Then, 10^5^ endothelial cells were treated with PBS, VEGF (40 μg/mL), Sema3A (50 μg/mL) and Sema3A (50 μg/mL) plus VEGF (40 μg/mL), and grown on matrigel coated plates at 37 °C with serum stavation. In addition, 10^5^ endothelial cells were also cultured in the matrigel with starvation medium from cultured SSC-9 with or without Sema3A overexpression. The Tubule structure was photographed 24 h after cell seeding by microscopy. Vascular cross points were counted in five randomly selected fields under a microscope in a blinded manner.

### Chick chorioallantoic membrane angiogenesis assay

Ten-day-old pathogen-free chick embryos were used for angiogenesis assays as previously described [20]. Briefly, 6 mm round filters were treated with PBS, VEGF165 (40 μg/mL), Sema3A (50 μg/mL) and Sema3A (50 μg/mL) plus VEGF165 (40 μg/mL). After the establishment of CAM, a filter was placed onto the non-vessel area of CAM. After 72 h of incubation, CAMs were harvested, and vascular density count was performed. Angiogenesis was quantified by counting the branch points arising from the tertiary vessels in a 6-mm-square area where the filter carrying the recombinant angiogenic factors was added. To count vessels, CAMs were photographed using a research stereoscope (model SZH10; Olympus, Melville, NY) equipped with a SPOT camera (model 2.2.1; Diagnostic Instruments, Sterling Heights, MI). PBS vessel count was subtracted as background.

### Animal model of tumor xenografts

Nude mice (male, 20-25 g) were used in this study. Tumor xenografts were generated by subcutaneous injection of 1 × 10^6^ cells into the flank of 6 week old BALB/cANNCjr nu/nu mice. Animals were monitored for 8 weeks, and sacrificed with high dose pentobarbital sodium (75 mg/kg). Tumors were harvested and weighted, and tumor dimensions were measured with calipers, and tumor volumes calculated using the simplified formula for a rotational ellipsoid: 0.4 × length^2^ × width.

### Capillary density measurement in oral cancer tissue samples

Tumors were harvested and snap-frozen in liquid nitrogen. Cryosections of 10 μm were prepared. Then, endothelial cells were stained with monoclonal anti-CD31 primary antibody (Pharmingen), followed by biotinylated anti-mouse IgG secondary antibody, and an avidin-HRP conjugate for color reaction (DAB paraffin IHC staining module, Ventana Medical Systems, Inc., Tucson, AZ). The sections were analyzed by microscopy, with 5 high power fields randomly selected in each section; CD31-positive cells were counted in a blinded manner. The number of CD 31-positive cells in each field was used as an index of capillary density.

### Statistical analysis

Data are mean ± standard deviation (SD). Student t-tests were performed to confirm significance of pairwise comparisons of vector control versus Sema 3A expressing cells. The other statistical comparisons were performed using ANOVA followed by Bonferroni/Dunn tests. *P* < 0.05 was considered statistically significant.

## Results

### Sema3A is expressed at low level in oral cancer

**Fig. 1:**
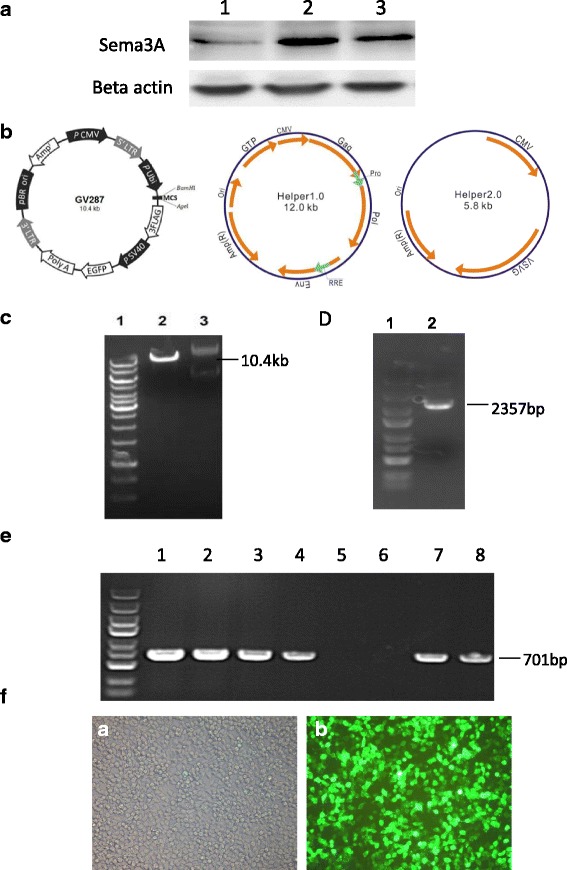
Sema3A expression in SSC-9 oral cancer line and construction of the LentiSema3A-EGFP vector. **a** The oral cancer cell line SSC-9 expresses lower Sema3A levels. Western blot was performed to detect protein expression of Sema3A in different cancer cell lines. Lane1, SCC-9; lane 2, R40LN; lane 3, R40P. The experiment was repeated 3 times. **b** Schematic structure of the backbone and helper plasmids for lentiviral vector construction. **c** Enzyme digestion of the backbone plasmid. Lane1, 10 Kb marker; lane2, AgeI digestion of the backbone plasmid; lane3, backbone plasmid without digestion. **d** PCR products of Sema3A. Lane1, 5 kb marker; lane2, PCR products of Sema3A with AgeI site at both ends. **e** PCR confirmation of successful ligation of backbone plasmid and Sema3A fragments. 1–8 are clones formed by ligation and transformed into *E. coli*. The results showed that 1–4 and 7,8 clones are correct. **f** Successful packaging of the Lentisema3A-EGFP vector. *a* 293 T cells under bright field; *b* 293 T cells under green fluorescent light. Magnification, ×100

Western blot was used to assess the protein expression levels of Sema3A in the oral cancer cell line SCC-9. The results showed that lower Sema3A levels were obtained in SCC-9 cells compared with R40LN and R40P cell lines (Fig. 1a).

### LentiSema3A transfection of oral cancer cells results in effective Sema3A overexpression

**Fig. 2:**
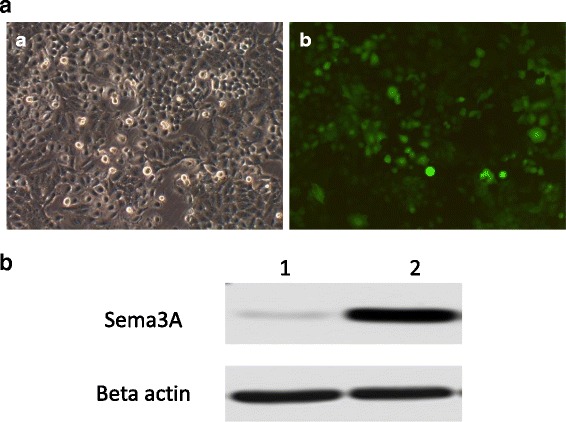
Lentiviral vector mediated efficient transduction of Sema3A in SSC-9 cells. **a** LentiSema3A-EGFP transfected into SSC-9 cells and Sema3A expression. *a* SSC-9 transfected with LentiSema3A-EGFP photographed under bright field; *b* SSC-9 transfected with LentiSema3A-EGFP photographed under green fluorescent light. Magnification, ×200. **b** Western blot analysis of Sema3A expression in SSC-9 cells transfected with LentiSema3A-EGFP. Lane 1: SSC-9 cells without Lentiviral transfection; Lane 2: SSC-9 cells with LentiSema3A-EGFP transfection

The lentiSema3A-EGFP vector was first successfully constructed (Fig. 1b–f) and transfected into oral cancer cells at 40 MOI. After 48 h of lentiviral transfection, GFP expression was observed in oral cancer cells; transduction efficiency reached almost 100% (Fig. 2a). The expression of Sema3A in oral cancer cells was further confirmed by Western blot. The results showed that Sema3A was efficiently expressed in oral cancer cells after lentiSema3A transfection (Fig. 2b).

### Sema3A inhibits vascular endothelial cell tubule formation

**Fig. 3:**
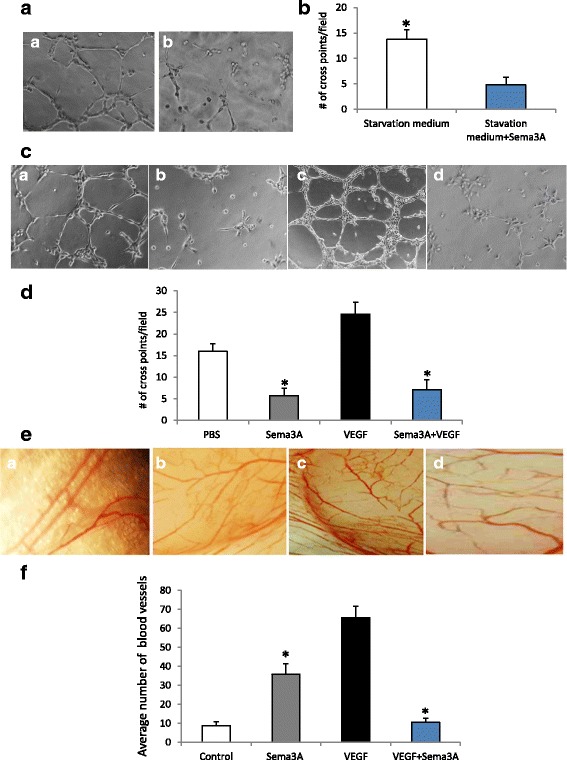
Sema3A inhibits VEGF-induced angiogenesis. **a**, *a* endothelial cells cultured with starvation medium from SSC-9; *b* endothelial cells cultured with starvation medium from SSC-9 with Sema3A overexpression. **b**, Sema3A significantly inhibits tubule formation of endothelial cells in the Matrigel cultured with starvation medium from SCC-9, compared with the control (*p* < 0.01). **c**, *a*: endothelial cells treated with PBS; *b* endothelial cells treated with Sema3A; *c* endothelial cells treated with VEGF; *d* endothelial cells treated with VEGF plus Sema3A. **d**, Sema3A significantly inhibits tubule formation of endothelial cells in the Matrigel caused by serum starvation, compared with PBS (*p* < 0.01); and Sema3A plus VEGF significantly inhibits tube formation of endothelial cells in the Matrigel, compared with VEGF (*p* < 0.01). **e**, *a* CAM treated with PBS; *b* CAM treated with Sema3A; *c* CAM treated with VEGF; *d* CAM treated with Sema3A + VEGF. **f**, Sema3A significantly increases angiogenesis compared with the control in CAM assay (*p* < 0.01); however, Sema3A plus VEGF significantly inhibits angiogenesis compared with the VEGF in CAM assay (*p* < 0.01)

The impact of Sema3A on endothelial cell tube formation was determined by the Matrigel gel assay. First, we showed that starvation medium from cultured SSC-9 with Sema3A overexpression significantly inhibited tubule formation in endothelial cells, (*P* < 0.01, Fig. 3a and b), which indicated Sema3A inhibited angiogenesis caused by tumor growth factors secreted from SSC-9. We further found that Sema3A significantly inhibited not only tubule formation of endothelial cells cultured with serum starvation, but also tubule formation of endothelial cells cultured with VEGF plus serum starvation (*P* < 0.01, Fig. 3c and d), which indicated that Sema3A inhibited VEGF-induced angiogenesis of endothelial cells.

### Sema3A inhibits angiogenesis in the chicken chorioallantoic membrane

Next, the effect of Sema3A on angiogenesis was assessed, using chicken chorioallantoic angiogenesis experiments. We found that Sema3A increased blood vessel numbers compared with the control, however, Sema3A plus VEGF significantly decreased blood vessel numbers in the chicken chorioallantoic membrane induced by VEGF (*P* < 0.01, Fig. 3e and f). This suggested that Sema3A inhibited VEGF-induced angiogenesis in CAM.

### Overexpression of Sema3A in oral cancer suppresses the growth of oral cancer by inhibiting angiogenesis

**Fig. 4:**
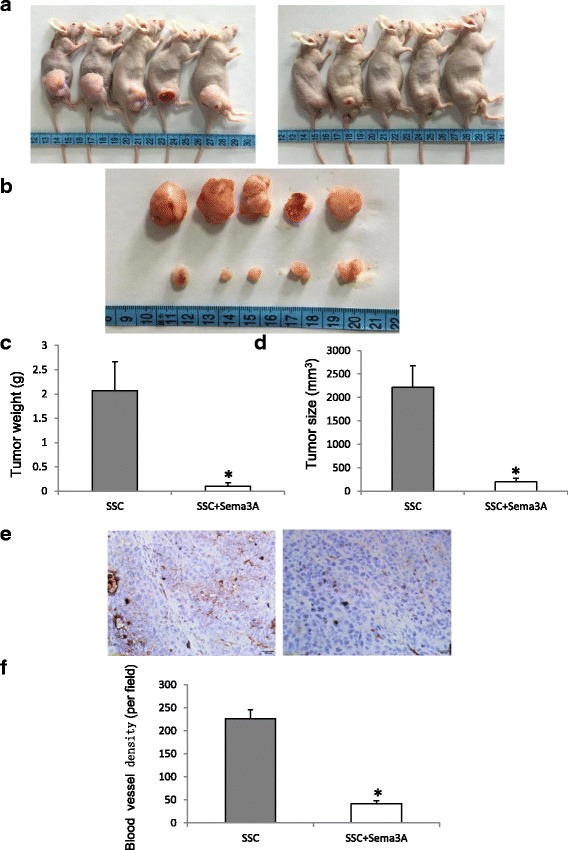
Overexpression of Sema3A in SSC-9 cells drastically suppresses tumor growth in vivo by inhibiting angiogenesis. **a** overexpression of Sema3A in SSC-9 cells inhibits tumor growth in vivo compared with the control, left panel: tumor with LentiSema-EGFP transfection; right panel: tumor with LentiSema3A-EGFP transfection. **b** upper panel: tumor with LentiSema-EGFP transfection; lower panel: tumor with LentiSema3A-EGFP transfection. **c** Sema3A significantly decreases tumor weight compared with the control (*p* < 0.01). **d** Sema3A significantly suppresses tumor growth compared with the control (*p* < 0.01). **e** immunostaining of tumor specimen with CD31 antibody, left panel: tumor with LentiSema-EGFP transfection; right panel: tumor with LentiSema3A-EGFP transfection. **f** Sema3A significantly decreases capillary density of tumor, bar = 100 μm, *p* < 0.01

To determine the effects of Sema3A on oral cancer growth, oral cancer cells overexpressing Sema3A were subcutaneously injected into nude mice. Animals were sacrificed at 8 weeks, and oral cancer specimens were harvested, measured and weighted. Interestingly, Sema3A drastically inhibited oral cancer growth in this xenograft model (Fig. 4a–d). Immunohistochemistry was performed to detect CD31 expression. The results showed that Sema3A significantly inhibited oral cancer angiogenesis (*P* < 0.01, Fig. 4e and f).

### Sema3A inhibits the phosphorylation of VEGFR2 as well as Src and FAK, downstream VEGF/VEGFR2

**Fig. 5:**
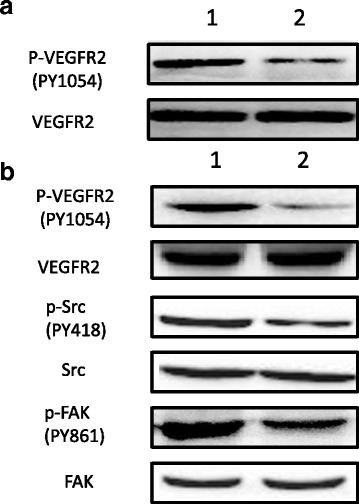
Overexpression of Sema3A in oral cancer inhibits VEGFR2 phosphorylation, and reduces the phosphorylation levels of its downstream genes Src and FAK. **a** lane1, VEGF-stimulating SSC-9 without Sema3A overexpression; lane2: VEGF-stimulating SSC-9 with Sema3A overexpression. **b** Lane 1: oral cancer treated with LentiEGFP; Lane 2: oral cancer treated with LentiSema3A-EGFP. The experiment was repeated 3 times

To determine the mechanism by which Sema3A inhibits angiogenesis, we focused on the effects of Sema3A on VEGFR2, and the subsequent effects on the VEGF/VEGFR2 downstream signaling pathway. The results showed that Sema3A inhibited the phosphorylation of VEGFR2; in addition, phosphorylation of the downstream genes Src and FAK in oral cancer was reduced, which results in tumor angiogenesis inhibition (Fig. 5).

## Discussion

Angiogenesis plays an important role in the formation, development, and metastasis of tumors [22]. Advances in modern tumor therapy suggest anti-angiogenesis to be a reasonable and promising treatment option for cancer [23]. In the present study, we found that overexpression of Sema3A in oral cancer cells using a lentiviral vector inhibited angiogenesis and drastically suppressed tumor growth, suggesting that Sema3A may be a potential target for the treatment of oral cancer in clinical practice.

Previous studies have shown that Sema3A inhibits angiogenesis in prostate cancer [24, 25]. In human squamous cell carcinoma of the tongue, Sema3A amounts are low, with higher expression levels prolonging the survival of patients with oral cancer [21]. We confirmed here that Sema3A is lowly expressed in the oral cancer cell line SSC-9. These findings indicated that Sema3A overexpression in oral cancer cells may also inhibit oral cancer growth by reducing angiogenesis. Interestingly, as shown above, Sema3A effectively inhibited tubular formation in endothelial cells as well as angiogenesis in chick embryo vascular assay. In addition, in vivo mouse subcutaneous xenograft experiment showed that lentiviral-mediated overexpression of Sema 3A in oral cancer cells drastically suppresses tumor growth, with angiogenesis overtly inhibited.

Sema3A is known to affect angiogenesis by suppressing integrins [26–29]. However, angiogenesis needs the coordinated effects of integrins and growth factor receptors [30]. Thus, Sema3A may play a role in the growth factor pathway. Indeed, a recent study found that Sema3A inhibits angiogenesis not only by suppressing integrins, but also affecting specific growth factors such as the VEGF signaling [20]. Since Sema3A and VEGF share a common receptor, Nrp-1, at least Sema3A could regulate the VEGF induced angiogenesis via Nrp-1. Nrp-1 plays an important role in the Sema3A mediated signaling pathway; indeed, Sema3A must bind to Nrp-1 before forming a complex with plexins [31]. Although Nrp-1 is not necessary for VEGF to play its role, it affects the function of the VEGF signaling pathway by altering VEGFR2 [32]. Therefore, Sema3A could affect VEGFR2, thereby altering angiogenesis through Nrp-1. In the present study, we demonstrated that Sema3A overexpression inhibits the phosphorylation of VEGFR2 in oral cancer tissues. In addition, the phosphorylation of Src and FAK, the downstream genes of VEGF/VEGFR2 were reduced as well, which results in angiogenesis inhibition. This is consistent with data reported by Acevedo et al. who found that Sema3A selectively inhibits VEGF-mediated angiogenesis [20].

Maione et al. further demonstrated that overexpression of Sema3A in tumor cells can normalize tumor vasculature and prevent its growth, eventually leaving the tumor in a steady state [19]. Because tumors can develop resistance toward the chemical drugs used against angiogenesis, Sema3A is highly significant to anti-tumor angiogenesis therapy. In fact, Sema3A was found to overcome drug resistance in pancreatic neuroendocrine tumors and cervical cancer to the small molecule tyrosine inhibitor imatinib [16]. Further studies are needed to further assess the relationship between Sema3A and chemotherapy targeting anti-angiogenesis in oral cancer.

Because tumor cells also express VEGFR2, in addition to inhibit angiogenesis, overexpression of Sema3A in oral cancer cells may inhibit tumor growth by direct effect to the tumor in our Xenograft model of oral cancer. Wang et al. also found that Sema3A inhibited growth of head and neck tumor by direct effect to the tumor cell in Xenograft model of mice [33]. Thus, Sema3A mediates inhibition of both tumorigenesis and angiogenesis in Xenograft oral cancer mode of mice. Recently, orthotopic model of oral cancer has been developed and used. Orthotopic model is better to simulate the anatomical environment of oral cancer, compared with Xenograft model of oral cancer. Further studies are needed to evaluate the effects of Sema3A on the oral cancer in orthotopic model.

## Conclusion

Overexpression of Sema3A in oral cancer cells drastically suppresses the growth of oral cancer by inhibiting angiogenesis. Sema3A reduces VEGFR2 phosphorylation in oral cancer, and further inhibits the phosphorylation of Src and FAK, downstream of VEGF/VEGFR2. These findings suggest that Sema3A could be used as an important target for the clinical treatment of oral cancer.

## Abbreviations

CAM assay: Chorioallantoic membrane assay
FAK: Focal adhesion kinase
HUVEC: Human umbilical vein endothelial cell
SCC-9: Squamous cell carcinoma
Sema3a: Semaphorin 3A
VEGF: Vascular endothelial growth factor
VEGFR2: Vascular endothelial growth factor receptor 2
